# Cross-sectional survey and analysis of factors influencing the prevalence of dental caries among older individuals aged 65-74 in Guangdong Province in 2021

**DOI:** 10.1186/s12903-024-04624-9

**Published:** 2024-08-16

**Authors:** Tao Tian, Zijing Yang, Simin Li, Weihua Fan, Linmei Wu, Jianbo Li, Shaohong Huang

**Affiliations:** Stomatological Hospital, School of Stomatology, Southern Medical University, Haizhu District, No. 366 Jiangnan South Avenue, Guangzhou, 510280 Guangdong Province China

**Keywords:** Guangdong Province, Aged 65-74, Dental caries, Cross-sectional, Epidemiology, Influencing factors

## Abstract

**Background:**

The prevalence of oral diseases is subject to change over time. In 2021, Guangdong Province conducted its fourth survey assessing the oral health status of individuals aged 65–74.

**Materials and methods:**

Evaluation criteria and potential influencing factors were identified. A sample of residents aged 65–74 from 13 designated monitoring sites in Guangdong Province was randomly selected for the study. Spearman correlation analysis was employed to investigate the clinical correlation between influencing factors and evaluation criteria. Negative binomial and zero-inflated negative binomial regression models were utilized to examine the factors influencing caries prevalence. In contrast, logistic regression was employed to identify the risk factors for caries occurrence. A *p*-value of ≤0.05 was considered statistically significant.

**Results:**

The prevalence of caries rate of crowns, roots, and teeth were 76.36%, 52.25%, and 79.2%, respectively. Individuals with periodontal pockets exhibited a significantly higher risk of root caries. The presence of dental calculus significantly exacerbated the occurrence of crown, root, and dental caries, and increased the risk of crown and dental caries. Consuming sweet foods once or more a week notably increased the average root decayed score (D of roots), the prevalence rate of root caries, and the D score of the Decayed, Missing, and Filled teeth [DMFT] index in individuals already afflicted with this condition. Similarly, the consumption of sweetened drinks significantly elevated the risk of crown and root caries, exacerbating overall caries progression. Frequencies of manual toothbrush and toothpick cleaning showed a negative correlation with average tooth missing score (MT). In contrast, the frequency of manual/electric toothbrush and toothpick cleaning was negatively correlated with the DMFT index. Engaging in dental diagnosis and treatment behaviors significantly increased the number of filled crowns (F), MT, and DMFT scores while reducing the prevalence of dental caries.

**Conclusions:**

In Guangdong Province, caries prevalence among older individuals aged 65–74 remains substantial. Relevant professionals and institutions must provide comprehensive guidance and assistance to the older population, emphasizing the importance of reducing the consumption of sweets and sweetened beverages, adopting correct tooth brushing techniques and frequency (at least twice daily), timely treatment of periodontal diseases, conducting regular epidemiological caries surveys, and addressing economic barriers to accessing caries diagnosis and treatment services.

## Background

As with other illnesses, oral diseases have significant emotional and psychosocial consequences [1]. In the 2017 global burden of disease rankings, oral diseases ranked among the most prevalent non-fatal health conditions [2]. Dental caries involve a progressive enamel lesion influenced by various oral factors, characterized by the demineralization of the inorganic component and degradation of the organic component [3]. It represents the most common chronic bacterial infection, presenting a substantial public health concern worldwide [4], affecting most adolescents and adults [5]. Despite being largely preventable, caries remains a significant global health challenge [6]. Estimates from 2015 indicated that approximately 2.5 billion people worldwide were affected by dental caries, impacting oral health [7].

Oral health indicate overall health and quality of life, particularly for older adults [1]. In recent decades, there has been significant progress in the oral health of the older population, marked by reduced prevalence of caries, periodontitis, and edentulism[8]. However, population-based surveys conducted in the United States of America (US) and Germany suggest that caries prevalence remains elevated among older adults [8]. The prevalence of oral diseases is subject to change over time[9], emphasizing the importance of conducting comprehensive oral epidemiological surveys every decade to monitor oral health trends [9]. Such surveys are crucial for researchers to elucidate current epidemiological characteristics and risk factors associated with oral diseases [9]. Data collected according to World Health Organization (WHO) standards facilitate comparisons between nations and regions [9]. Moreover, research findings serve as a scientific foundation for dental public health practitioners and practicing dentists to develop effective preventive strategies [9].

According to the seventh National Census, Guangdong Province boasts a population of 126 million, making it the most populous province in China. The population growth in Guangdong Province has influenced dietary habits, oral health behaviors, population demographics, and economic development [9], thereby impacting oral health outcomes. Understanding the factors driving poor oral health and oral health disparities is crucial for formulating appropriate policies and interventions [10]. This research employed epidemiological methods to investigate the prevalence and analyze the factors that influence the prevalence of crown and root caries among the older population aged 65–74 years in Guangdong Province in 2021.

## Materials and methods

### Ethics related

Before conducting the survey, it was reviewed and approved by the Stomatological Ethics Committee of the Chinese Stomatological Association (Approval No.: 2014–003).

### Survey participants

Following the rapid oral health survey method established by the WHO, the survey targeted older individuals aged 65–74 years residing in the permanent population at 13 disease monitoring points in Guangdong Province, as designated by the National Project Office through sampling. Inclusion criteria comprised individuals aged 65–74 years, residents of Guangdong Province (with a minimum residency of 6 months before the survey), and voluntary participation. Exclusion criteria encompassed individuals with severe systemic diseases (such as cardiovascular, digestive, respiratory, blood, and neurological diseases), mental illnesses, and those unable to achieve adequate mouth opening for examination. Before participation, all survey participants provided informed consent by signing a consent form.

### Sample design

**Fig. 1:**
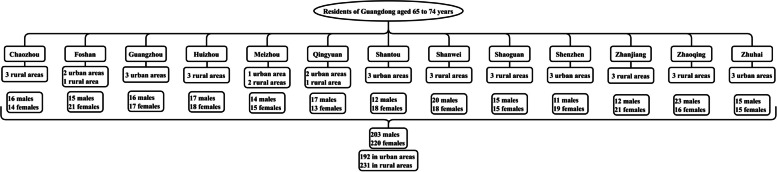
The number of people surveyed at each monitoring point

Following the principles of stratified random sampling, oral health monitoring was conducted at 13 disease monitoring points across Guangdong Province. The sampling approach was community-based, with three village (neighborhood) committees selected as survey units at each monitoring point. Within each survey unit, 9–14 residents aged 65–74 were randomly chosen for participation. The breakdown of surveyed individuals at each monitoring point is illustrated in Fig 1.

### Survey contents and methods

#### Selection and training, quality control of investigators

Specific qualifications were required for oral examiners, including being licensed practicing dentists with a minimum of 3 years of clinical experience in dentistry. Recorders could be physicians or nurses with some clinical experience in dentistry. Each monitoring point was equipped with two examiners and two recorders. Examiners underwent provincial-level training to ensure proficiency. A Kappa value of ≥ 0.8 was considered completely reliable for assessing caries status, while a Kappa value of ≥0.6 indicated good reliability for periodontal pocket depth. Participants underwent re-examination by a different examiner at a 5% re-examination rate. Re-examinations focused on half of the dentition, with participants assigned to odd or even-numbered quadrants based on their ID number’s ending digit (such as the first and third quadrants, upper right/lower left, or the second and fourth quadrants, upper left/lower right). All re-examination results were preserved and analyzed for standard consistency with initial examination findings. Throughout the survey, the provincial expert group re-examined five participants for each examiner, following the same procedure as independent examiner re-examinations. The calculated Kappa values from all re-examination results had to meet established standards.

Questionnaire surveyors comprised oral medical and nursing staff or public health personnel who were willing to participate and had adept social communication skills. Two surveyors were stationed at each monitoring point, all of whom underwent standardized provincial training and assessment. A questionnaire consistency rate exceeding 90% was required for qualification.

#### Dental examination

The examination was conducted under artificial light, combining visual inspection and probing. The examination instruments included flat mouth mirrors and Community Periodontal Index (CPI) probes; soft deposits were removed with a cotton swab when necessary. The examination proceeded in a specific order, starting from the third permanent molar in the upper right quadrant, moving to the third permanent molar in the upper left quadrant, then to the third permanent molar in the lower left quadrant, and finally to the third permanent molar in the lower right quadrant. Each tooth or missing tooth space should be examined individually, including the third molars. The diagnostic criteria used in this study adhered to the Basic Methods for Oral Health Surveys by WHO (5th Edition).

Decayed (D): Crown decay was recorded if there was a clear cavity in the crown, an apparent sub-enamel destruction, or a distinctly palpable softened base or wall of the cavity. Root decay was recorded when the CPI probe detected destruction of the root surface dentin, feeling soft or leathery. If a carious lesion simultaneously affected both the crown and root of a tooth, it was recorded as both crown and root decay, and the tooth was defined as decay.

Missing (M): Any tooth lost due to decay or any other cause was classified as "missing."

Filled (F): A tooth was considered “filled” if its crown or root had fillings, provided there was no decay on the same part of the tooth.

Periodontal pocket: A periodontal pocket was defined as having a probing depth of ≥4mm around the dental periphery.

Dental calculus: If calculus was detected when probing, it was recorded as dental calculus.

#### Questionnaire survey

For data collection, participants were interviewed face-to-face by questionnaire surveyors at the oral health examination site. This study included the following variables in the questionnaire: (1) Gender and age. (2) Socio-economic status, including residential area, educational level, and annual family income. (3) Personal lifestyle factors, including intake of sweets, sugary drinks, smoking status, and number of persons living together. (4) Personal health status, including the presence of systemic diseases and major organ diseases. (5) Dental care behaviors, including methods and frequency of teeth cleaning, and whether the participant had received dental diagnosis and treatment at a medical institution.

#### Evaluation indicators and related influencing factors

Evaluation indicators

$$\text{D of crowns }\left(\text{Average crown decayed score}\right)=\frac{\text{Sum of decayed crown among surveyed individual}}{\text{Sum of individual surveyed}};$$

$$\text{F of crowns }\left(\text{Average crown filled score}\right)= \frac{\text{Sum of filled crown among surveyed individual}}{\text{Sum of individual surveyed}};$$

$$\text{DF of crowns }\left(\text{Average crown decayed and filled score}\right)= \frac{\text{Sum of decayed and filled crown among individual}}{\text{Sum of individual surveyed}};$$

$$\text{D of roots }\left(\text{Average root decayed score}\right)= \frac{S\text{um of decayed root among surveyed individual}}{\text{Sum of individual surveyed}};$$

$$\text{F of roots }\left(\text{Average root filled score}\right)= \frac{\text{Sum of filled root among surveyed individual}}{\text{Sum of individual surveyed}};$$

$$\text{DF of roots }\left(\text{Average root decayed and filled score}\right)= \frac{\text{Sum of decayed and filled root among surveyed individual}}{\text{Sum of individual surveyed}};$$

$$\text{D of teeth }\left(\text{Average tooth decayed score}\right)= \frac{\text{Sum of decayed tooth among surveyed individual}}{\text{Sum of individual surveyed}};$$

$$\text{F of teeth }\left(\text{Average tooth filled score}\right)= \frac{\text{Sum of filled tooth among surveyed individual}}{\text{Sum of individual surveyed}};$$

$$\text{DFT }\left(\text{Average tooth decayed and filled score}\right)= \frac{\text{Sum of decayed and filled tooth among surveyed individual}}{\text{Sum of individual surveyed}};$$

$$\text{MT }\left(\text{Average tooth missing score}\right)= \frac{\text{Sum of missing tooth among surveyed individual}}{\text{Sum of individual surveyed}};$$

$$\text{DMFT }\left(\text{Average tooth decayed},\text{ missing},\text{ and filled score}\right)= \frac{\text{Sum of decayed},\text{ missing and filled tooth among surveyed individual}}{\text{Sum of individual surveyed}};$$

$$\text{Coronary caries rate}=\frac{\text{Sum of carious crown among surveyed individual}}{\text{Sum of individual surveyed}}\text{x}100\%;$$

$$\text{Root caries rate}=\frac{\text{Sum of carious root among surveyed individual}}{\text{Sum of individual surveyed}}\text{x}100\%;$$

$$\text{Dental caries rate}=\frac{S\text{um of carious tooth among surveyed individual}}{\text{Sum of individual surveyed}}\text{x}100\%.$$

#### Influencing factors

The related influencing factors were divided into various groups:

1. Gender: Men and women; 2. Age: 65–69 and 70–74 years; 3. Periodontal pocket: With and Without; 4. Dental calculus: With and Without; 5. Residential area: Urban and Rural; 6. Education level: Illiterate, Primary school, Junior high school, Senior high school and above; 7. Family income in the last year (×10,000 RMB): 1–3, 4–9, 10–30, and prefer not to disclose; 8. Frequency of consuming sweet snacks and candies: Never, 1–3 times/month, and once or more than once a week; 9. Frequency of consuming sweet drinks: Never and once or more than once a month; 10. Frequency of consuming beverages such as sugary milk, yogurt, tea, soy milk, coffee, and milk tea: Never, 1–4 times/month, and twice or more than twice a week; 11. Smoking status: Non-smoker, Ex-smoker, and Smoker; 12. Smoking duration (years): 1–36, 40–49, and 50–60; 13. Number of people living together in the household: 1–3, 4 or 5, 6–17, and prefer not to disclose; 14. Hypertension: Yes and No; 15. Stroke: Yes and No; 16. Diabetes: Yes and No; 17. Coronary heart disease: Yes and No; 18. Frequency of cleaning teeth with manual toothbrushes (times/day): <1, 1, and ≥2; 19. Frequency of cleaning teeth with electric toothbrushes: Never and once or more than once a month; 20. Frequency of cleaning teeth with toothpicks: Never, once a month to once a day, and twice or more than twice a day; 21. Frequency of using dental floss: Never and once or more than once a month; 22. Frequency of using interdental brushes: Never and once or more than once a month; 23. Frequency of using water floss: Never and once or more than once a month; 24. Frequency of using commercial mouthwash: Never and once or more than once a month; 25. Routine use of fluoride toothpaste: Yes and No; 26. Visit to a medical institution for dental diagnosis and treatment: Yes and No.

### Statistical analysis

#### Data stratification and statistical criteria for significant differences

The data were organized in ascending order based on factors such as age, educational level, income, frequency, number of individuals, and disease severity. The group with the lowest median value or the least severe disease was designated as the reference group. Within the gender and residential area categories, the male and rural groups were respectively set as reference groups. The non-smoking group was set as the reference group for the smoking category, with the ex-smoking and smoking groups following in ascending order. Dummy variable 1 represented the ex-smoking group relative to the reference group, while dummy variable 2 represented the smoking group relative to the reference group. Statistical significance was set at a *p*-value of ≤0.05.

#### Cross-sectional survey of caries prevalence

Spearman correlation analysis was used to explore the clinical relevance between related influencing factors and evaluation indicators.

#### Analysis of related influencing factors on caries

The Poisson regression model is commonly used in count data models. However, Poisson regression theoretically requires that the mean and variance are equal. If this condition is not met, the negative binomial regression model can be utilized as it relaxes the assumption that the mean equals the variance. In practical research, a scenario may occur where the dependent variable is a count variable with numerous zeros. In such cases, the zero-inflated negative binomial regression model can be applied. This model presents two parts: "Count model" indicates the non-zero count part, referring to the effect of independent variables on the quantity of the dependent variable; "Zero-inflation model" indicates the zero-count part, referring to the impact of independent variables on whether the dependent variable occurs or not.

In multivariate analysis, logistic regression analysis was used to identify the risk factors for the prevalence of caries.

## Results

### Cross-sectional prevalence of caries

The indicators of caries prevalence: crowns: D=2.6, F=0.74, DF=3.35; roots: D=1.62, F=0.13, DF=1.75; teeth: D=2.98, F=0.76, DFT=3.74; MT=6.61; DMFT=10.34; Coronary caries rate=76.36%; root caries rate=52.25%; dental caries rate=79.2%.

The results of the Spearman correlation analysis for the related influencing factors on evaluation indicators are shown in Table 1.

**Table 1:** Spearman correlation analysis of influencing factors on evaluation indicators

**Independent variable** **Dependent variable**		**Gender**	**Periodontal pocket**	**Dental calculus**	**Residential area**	**Educational leve**	**Income in last year**	**Sugary snack and candy**	**Sweet drink**	**Sugary drink**
**D of crowns**	r	0.065	0.004	0.003	-0.083	-0.013	-0.097	-0.025	0.125	0.132
	*p*	0.184	0.933	0.952	0.088	0.792	**0.046**	0.602	**0.01**	**0.006**
**F of crowns**	r	0.162	-0.034	0.035	0.315	0.091	-0.093	0.159	0.062	0.1
	*p*	**0.001**	0.486	0.478	**<0.001**	0.061	0.056	**0.001**	0.206	**0.04**
**DF of crowns**	r	0.138	-0.008	0.005	0.081	0.029	-0.115	0.04	0.128	0.163
	*p*	**0.005**	0.875	0.913	0.095	0.546	**0.018**	0.414	**0.009**	**0.001**
**D of roots**	r	0.096	0.126	-0.039	0.047	0.018	-0.085	0.019	0.072	0.132
	*p*	**0.047**	**0.01**	0.418	0.338	0.716	0.082	0.693	0.141	**0.007**
**F of roots**	r	0.072	0.026	0.123	0.071	0.056	0.024	0.052	-0.013	-0.05
	*p*	0.142	0.598	**0.011**	0.144	0.252	0.624	0.286	0.797	0.306
**DF of roots**	r	0.099	0.123	-0.024	0.064	0.027	-0.074	0.028	0.065	0.116
	*p*	**0.043**	**0.011**	0.623	0.189	0.578	0.129	0.563	0.182	**0.017**
**D of teeth**	r	0.071	0.021	0.004	-0.101	0.01	-0.067	-0.021	0.112	0.149
	*p*	0.146	0.671	0.938	**0.039**	0.842	0.171	0.665	**0.022**	**0.002**
**F of teeth**	r	0.152	-0.041	0.043	0.31	0.099	-0.087	0.153	0.055	0.098
	*p*	**0.002**	0.397	0.373	**<0.001**	**0.042**	0.073	**0.002**	0.261	**0.044**
**DFT**	r	0.135	0	0.001	0.056	0.046	-0.084	0.045	0.112	0.181
	*p*	**0.005**	0.997	0.981	0.25	0.344	0.083	0.36	**0.021**	**<0.001**
**MT**	r	0.043	-0.028	-0.343	0.087	0.032	-0.094	0.123	0.082	0.174
	*p*	0.376	0.559	**<0.001**	0.074	0.511	0.053	**0.011**	0.091	**<0.001**
**DMFT**	r	0.07	-0.067	-0.33	0.07	0.073	-0.128	0.144	0.13	0.245
	*p*	0.153	0.17	**<0.001**	0.153	0.133	**0.009**	**0.003**	**0.008**	**<0.001**
**Coronary caries rate**	r	0.03	0.004	-0.019	-0.063	-0.004	-0.093	-0.027	0.162	0.105
	*p*	0.537	0.936	0.69	0.199	0.94	0.057	0.582	**0.001**	**0.031**
**Root caries rate**	r	0.082	0.147	-0.003	0.076	0.028	-0.095	-0.002	0.043	0.114
	*p*	0.094	**0.003**	0.944	0.116	0.561	0.052	0.966	0.372	**0.019**
**Dental caries rate**	r	0.068	0.019	-0.02	-0.075	0.024	-0.072	-0.019	0.139	0.083
	*p*	0.161	0.698	0.689	0.122	0.622	0.141	0.697	**0.004**	0.089
**D of crowns**	r	-0.133	0.054	0.051	-0.098	-0.105	0.109	0.011	0.089	-0.082
	*p*	**0.006**	0.267	0.295	**0.044**	**0.031**	**0.025**	0.817	0.067	0.093
**F of crowns**	r	-0.217	-0.191	0.035	0.218	-0.003	0.001	0.163	0.042	0.29
	*p*	**<0.001**	**<0.001**	0.477	**<0.001**	0.949	0.981	**0.001**	0.39	**<0.001**
**DF of crowns**	r	-0.212	-0.042	0.061	0.003	-0.107	0.087	0.087	0.108	0.048
	*p*	**<0.001**	0.394	0.214	0.957	**0.028**	0.073	0.074	**0.026**	0.329
**D of roots**	r	-0.132	0.048	0.029	-0.086	-0.093	0.009	-0.026	0.08	0.004
	*p*	**0.006**	0.325	0.547	0.078	0.055	0.859	0.589	0.1	0.936
**F of roots**	r	-0.026	-0.038	0.164	0.083	-0.033	0.052	0.069	-0.026	0.089
	*p*	0.592	0.440	**0.001**	0.09	0.499	0.288	0.156	0.598	0.067
**DF of roots**	r	-0.125	0.038	0.056	-0.065	-0.097	0.018	-0.017	0.076	0.016
	*p*	**0.01**	0.434	0.254	0.184	**0.047**	0.709	0.727	0.12	0.739
**D of teeth**	r	-0.13	0.071	0.058	-0.097	-0.112	0.084	-0.008	0.089	-0.046
	*p*	**0.008**	0.146	0.235	**0.045**	**0.021**	0.084	0.869	0.069	0.347
**F of teeth**	r	-0.208	-0.192	0.031	0.214	-0.005	-0.007	0.16	0.04	0.292
	*p*	**<0.001**	**<0.001**	0.525	**<0.001**	0.926	0.891	**0.001**	0.407	**<0.001**
**DFT**	r	-0.203	-0.022	0.062	-0.001	-0.114	0.066	0.061	0.103	0.069
	*p*	**<0.001**	0.650	0.205	0.985	**0.018**	0.175	0.207	**0.034**	0.156
**MT**	r	0	-0.008	0.004	-0.025	-0.042	-0.061	-0.034	0.026	0.172
	*p*	0.997	0.875	0.935	0.614	0.393	0.211	0.492	0.588	**<0.001**
**DMFT**	r	-0.074	0.007	0.032	-0.032	-0.089	-0.08	-0.014	0.05	0.175
	*p*	0.127	0.882	0.516	0.511	0.067	0.102	0.776	0.303	**<0.001**
**Coronary caries rate**	r	-0.119	-0.101	0.029	-0.051	-0.071	0.103	0.057	0.067	-0.055
	*p*	**0.014**	**0.037**	0.555	0.294	0.142	**0.034**	0.24	0.168	0.256
**Root caries rate**	r	-0.112	0.009	0.037	-0.064	-0.101	0.028	0.033	0.065	0.034
	*p*	**0.021**	0.849	0.444	0.191	**0.037**	0.566	0.494	0.181	0.489
**Dental caries rate**	r	-0.144	-0.059	0.036	-0.05	-0.067	0.074	0.026	0.085	0.005
	*p*	**0.003**	0.225	0.46	0.305	0.166	0.13	0.591	0.081	0.914

### Analysis of influencing factors on caries

The analysis results of the influencing factors on D, F, and DF of crowns, D and DF of roots, D and F of teeth, DFT, MT, and DMFT are shown in Tables 2, 3, 4, 5, 6, 7, 8, 9, 10, 11, respectively. The analysis results for the factors influencing the risk of caries in crowns, roots, and teeth are shown in Tables 12, 13, 14, respectively. Due to the zero count of the negative binomial regression for F of roots being 96.69% (409/423), the calculation cannot be performed.

**Table 2:** Negative binomial regression analysis of the influencing factors on D of crowns

	**IRR**	**lower**	**upper**	**Z**	***p***
**(Intercept)**	1.534	1.041	2.261	2.163	0.031
**Dental calculus**	2.113	1.475	3.026	4.079	<0.001
**Sweet drink**	1.354	1.07	1.714	2.520	0.012
**Smoking 1**	0.991	0.686	1.433	-0.046	0.963
**Smoking 2**	0.638	0.483	0.842	-3.175	0.001
**Manual toothbrush**	0.7	0.553	0.885	-2.986	0.003
**Electric toothbrush**	0.271	0.12	0.616	-3.119	0.002

**Table 3:** Negative binomial regression analysis of the influencing factors on F of crowns

	**IRR**	**lower**	**upper**	**Z**	***p***
**(Intercept)**	0.146	0.073	0.291	-5.447	<0.001
**Residential area**	3.44	2.053	5.765	4.69	<0.001
**Smoking 1**	0.669	0.341	1.311	-1.17	0.242
**Smoking 2**	0.233	0.115	0.471	-4.056	<0.001
**Person living together 1**	0.713	0.419	1.212	-1.251	0.211
**Person living together 2**	0.548	0.301	0.996	-1.974	0.048
**Person living together 3**	0.831	0.299	2.312	-0.354	0.723
**Manual toothbrush**	1.598	1.004	2.544	1.976	0.048
**Dental diagnosis and treatment**	3.615	2.085	6.27	4.575	<0.001

**Table 4:** Zero-inflated negative binomial regression analysis of the influencing factors on DF of crowns

**Count model**						**Zero-inflation model**					
	**IRR**	**lower**	**upper**	**Z**	***p***		**IRR**	**lower**	**upper**	**Z**	***p***
**(Intercept)**	3.4	2.931	3.945	16.15	<0.001	**(Intercept)**	0.142	0.032	0.642	-2.536	0.011
**Sweet drink**	1.347	1.096	1.656	2.829	0.005	**Dental calculus**	0.014	0.000	0.501	-2.339	0.019
**Smoking 1**	1.235	0.865	1.762	1.161	0.246	**Smoking 1**	30.9	2.414	395.605	2.637	0.008
**Smoking 2**	0.629	0.479	0.825	-3.346	0.001	**Smoking 2**	13.518	2.174	84.042	2.793	0.005
**Log(theta)**	1.39	1.079	1.789	2.552	0.011	**Electric toothbrush**	80.864	1.72	3802.409	2.236	0.025

**Table 5:** Zero-inflated negative binomial regression analysis of the influencing factors on D of roots

Count model						Zero-inflation model					
	IRR	lower	upper	Z	*p*		IRR	lower	upper	Z	*p*
(Intercept)	0.867	0.517	1.454	-0.542	0.588	(Intercept)	0.235	0.026	2.113	-1.293	0.196
Age	1.509	1.147	1.986	2.939	0.003	Educational level 1	0.106	0.019	6.02e-01	-2.534	0.011
Dental calculus	2.041	1.323	3.15	3.224	0.001	Educational level 2	0.028	0.002	3.5e-01	-2.773	0.006
Sugary snack and candy 1	1.326	0.943	1.866	1.621	0.105	Educational level 3	0.02	0.002	2.5e-01	-3.035	0.002
Sugary snack and candy 2	2.55	1.71	3.802	4.593	<0.001	Sugary snack and candy 1	0.81	0.047	1.4007e+01	-0.145	0.885
Smoking 1	0.811	0.482	1.367	-0.786	0.432	Sugary snack and candy 2	24.973	1.798	3.46838e+02	2.397	0.017
Smoking 2	0.474	0.338	0.665	-4.328	<0.001	Smoking 1	12.989	2.215	7.6174e+01	2.841	0.004
Manual toothbrush	0.537	0.402	0.719	-4.182	<0.001	Smoking 2	0.003	0.000	4.393135e+17	-0.247	0.805
Electric toothbrush	0.286	0.103	0.699	-2.691	0.007						
Log(theta)	0.988	0.694	1.407	-0.066	0.948

**Table 6:** Zero-inflated negative binomial regression analysis of the influencing factors on DF of roots

**Count model**						**Zero-inflation model**					
	**IRR**	**lower**	**upper**	**Z**	***p***		**IRR**	**lower**	**upper**	**Z**	***p***
**(Intercept)**	0.885	0.493	1.588	-0.41	0.682	**(Intercept)**	0.22	0.066	0.73	-2.473	0.013
**Age**	1.622	1.224	2.149	3.366	0.001	**Electric toothbrush**	7.979	1.31	48.611	2.253	0.024
**Dental calculus**	1.916	1.237	2.967	2.912	0.004						
**Educational level 1**	1.321	0.879	1.985	1.341	0.18						
**Educational level 2**	1.94	1.221	3.083	2.807	0.005						
**Educational level 3**	1.684	1.031	2.751	2.082	0.037						
**Smoking 1**	0.695	0.426	1.134	-1.455	0.146						
**Smoking 2**	0.563	0.397	0.798	-3.222	0.001						
**Manual toothbrush**	0.687	0.511	0.925	-2.478	0.013						
**Log(theta)**	0.972	0.532	1.777	-0.092	0.927

**Table 7:** Negative binomial regression analysis of the influencing factors on D of teeth

	**IRR**	**lower**	**upper**	**Z**	***p***
**(Intercept)**	1.608	1.077	2.401	2.322	0.02
**Dental calculus**	1.943	1.395	2.707	3.926	<0.001
**Educational level 1**	1.274	0.942	1.723	1.571	0.116
**Educational level 2**	1.52	1.069	2.161	2.333	0.02
**Educational level 3**	1.311	0.902	1.906	1.419	0.156
**Sweet drink**	1.273	1.021	1.589	2.141	0.032
**Smoking 1**	0.876	0.614	1.249	-0.732	0.464
**Smoking 2**	0.601	0.46	0.784	-3.743	<0.001
**Manual toothbrush**	0.658	0.524	0.827	-3.583	<0.001
**Electric toothbrush**	0.283	0.133	0.599	-3.298	0.001

**Table 8:** Negative binomial regression analysis of the influencing factors on F of teeth

**Count model**						**Zero-inflation model**					
	**IRR**	**lower**	**upper**	**Z**	***p***		**IRR**	**lower**	**upper**	**Z**	***p***
**(Intercept)**	0.581	0.334	1.013	-1.916	0.055	**(Intercept)**	3.31	1.429	7.667	2.793	0.005
**Residential area**	4.681	2.973	7.371	6.663	<0.001	**Dental diagnosis and treatment**	0.117	0.035	0.39	-3.489	<0.001
**Smoking 1**	1.025	0.521	2.018	0.073	0.942						
**Smoking 2**	0.243	0.125	0.473	-4.154	<0.001						
**Log(theta)**	0.811	0.292	2.256	-0.401	0.688

**Table 9:** Zero-inflated negative binomial regression analysis of the influencing factors on DFT

**Count model**						**Zero-inflation model**					
	**IRR**	**lower**	**upper**	**Z**	***p***		**IRR**	**lower**	**upper**	**Z**	***p***
**(Intercept)**	3.278	2.589	4.15	9.864	<0.001	**(Intercept)**	0.111	0.021	0.58	-2.606	0.009
**Educational level 1**	1.166	0.895	1.52	1.137	0.256	**Dental calculus**	0.026	0.002	0.294	-2.945	0.003
**Educational level 2**	1.406	1.038	1.905	2.201	0.028	**Smoking 1**	15.632	2.334	104.681	2.834	0.005
**Educational level 3**	1.242	0.904	1.706	1.334	0.182	**Smoking 2**	20.132	2.104	192.611	2.606	0.009
**Sweet drink**	1.266	1.043	1.537	2.382	0.017	**Electric toothbrush**	63.001	3.57	1111.919	2.829	0.005
**Smoking 1**	1.122	0.799	1.575	0.666	0.505						
**Smoking 2**	0.644	0.5	0.829	-3.41	0.001						
**Log(theta)**	1.592	1.23	2.061	3.53	<0.001

**Table 10:** Negative binomial regression analysis of the influencing factors on MT

	**IRR**	**lower**	**upper**	**Z**	***p***
**(Intercept)**	11.123	7.941	15.58	14.013	<0.001
**Dental calculus**	0.496	0.388	0.634	-5.597	<0.001
**Educational level 1**	1.356	1.057	1.741	2.392	0.017
**Educational level 2**	1.394	1.05	1.849	2.3	0.021
**Educational level 3**	1.087	0.804	1.469	0.542	0.588
**Manual toothbrush**	0.812	0.675	0.978	-2.198	0.028
**Toothpick 1**	0.695	0.542	0.891	-2.866	0.004
**Toothpick 2**	0.699	0.561	0.871	-3.187	0.001
**Dental diagnosis and treatment**	1.287	1.066	1.555	2.619	0.009

**Table 11:** Negative binomial regression analysis of the influencing factors on DMFT

	IRR	lower	upper	Z	*p*
(Intercept)	12.178	9.321	15.913	18.318	<0.001
Dental calculus	0.679	0.562	0.819	-4.04	<0.001
Educational level 1	1.237	1.025	1.492	2.22	0.026
Educational level 2	1.37	1.106	1.696	2.887	0.004
Educational level 3	1.109	0.883	1.391	0.89	0.374
Sugary snack and candy 1	1.129	0.959	1.329	1.457	0.145
Sugary snack and candy 2	1.24	1.039	1.481	2.387	0.017
Manual toothbrush	0.814	0.707	0.937	-2.861	0.004
Electric toothbrush	0.497	0.327	0.754	-3.282	0.001
Toothpick 1	0.835	0.692	1.007	-1.889	0.059
Toothpick 2	0.786	0.664	0.93	-2.808	0.005
Dental diagnosis and treatment	1.217	1.053	1.406	2.662	0.008

**Table 12:** Logistic regression analysis of risk factors for prevalence of crown caries

**B**	**B**	**Standard error**	**Wald**	**Degree of freedom**	**Significance**	**Exp(β)**	**95% confidence interval of Exp (B)**	
							**Lower limit**	**Upper limit**
**Dental calculus**	1.281	0.319	16.089	1	<0.001	3.602	1.926	6.736
**Sweet drink**	0.606	0.273	4.912	1	0.027	1.833	1.073	3.133
**Smoking**			18.123	2	<0.001			
**Smoking 1**	-0.677	0.389	3.033	1	0.082	0.508	0.237	1.089
**Smoking 2**	-1.154	0.274	17.791	1	<0.001	0.315	0.184	0.539
**Electric toothbrush**	-1.685	0.632	7.106	1	0.008	0.185	0.054	0.64
**Toothpick**			8.633	2	0.013			
**Toothpick 1**	0.852	0.343	6.155	1	0.013	2.344	1.196	4.593
**Toothpick 2**	0.772	0.293	6.956	1	0.008	2.164	1.219	3.842
**Constant**	-0.188	0.353	0.284	1	0.594	0.829

**Table 13:** Logistic regression analysis of risk factors for prevalence of root caries

	**B**	**Standard error**	**Wald**	**Degree of freedom**	**Significance**	**Exp(β)**	**95% confidence interval of Exp (B)**	
							**Lower limit**	**Upper limit**
**Periodontal pocket**	0.794	0.224	12.511	1	<0.001	2.211	1.425	3.432
**Residential area**	0.533	0.225	5.634	1	0.018	1.704	1.097	2.646
**Educational level**			6.637	3	0.084			
**Educational level 1**	0.664	0.291	5.195	1	0.023	1.942	1.097	3.436
**Educational level 2**	0.795	0.34	5.475	1	0.019	2.214	1.138	4.307
**Educational level 3**	0.586	0.356	2.714	1	0.099	1.798	0.895	3.611
**Smoking**			8.597	2	0.014			
**Smoking 1**	-0.728	0.347	4.406	1	0.036	0.483	0.245	0.953
**Smoking 2**	-0.629	0.253	6.201	1	0.013	0.533	0.325	0.875
**Manual toothbrush 2**	-0.649	0.226	8.229	1	0.004	0.523	0.336	0.814
**Electric toothbrush**	-1.903	0.696	7.484	1	0.006	0.149	0.038	0.583
**Constant**	-0.672	0.301	4.98	1	0.026	0.511

**Table 14:** Logistic regression analysis of risk factors for prevalence of tooth caries

	**B**	**Standard error**	**Wald**	**Degree of freedom**	**Significance**	**Exp(β)**	**95% confidence interval of Exp (B)**	
							**Lower limit**	**Upper limit**
**Dental calculus**	1.154	0.315	13.412	1	<0.001	3.172	1.71	5.884
**Smoking**			11.861	2	0.003			
**Smoking 1**	-0.666	0.395	2.847	1	0.092	0.514	0.237	1.114
**Smoking 2**	-0.94	0.28	11.255	1	0.001	0.391	0.226	0.677
**Electric toothbrush**	-1.598	0.602	7.058	1	0.008	0.202	0.062	0.658
**Toothpick**			5.744	2	0.057			
**Toothpick 1**	0.825	0.361	5.224	1	0.022	2.281	1.125	4.626
**Toothpick 2**	0.536	0.298	3.226	1	0.072	1.709	0.952	3.068
**Constant**	0.357	0.334	1.139	1	0.286	1.428		

Dental calculus increased the D of crowns and teeth, D and DF of roots, and the risk of crown and tooth caries while significantly reducing MT, DMFT, and the likelihood of DF of crowns >0 and DFT >0. Periodontal pockets significantly increased the risk of root caries. Aging significantly increased the D and DF of roots. Urban residents had significantly higher F of crowns and teeth, and a higher risk of root caries than rural residents. Higher education levels increased DF of roots and reduced the likelihood of D of roots >0. Junior high school education increased D of teeth and DFT, while primary and junior high school education significantly increased MT, DMFT, and the risk of root caries. The intake of sugary snacks and candies once or more than once a week substantially increased D of roots, DMFT, and the likelihood of D of roots >0. Sweet drink intake considerably increased D of crowns and teeth, DF of crowns, DFT, and the risk of crown caries. Smoking significantly reduced D and F of crowns and teeth, DF of crowns, D and DF of roots, DFT, and the risk of crown and tooth caries while increasing the likelihood of DFT >0. Those who quit smoking increased the possibility of D of roots >0. When the number of people living together in a household was 6 to 17, it significantly reduced F of crowns. Manual toothbrushing significantly reduced D of crowns and DMFT, with manual toothbrushing twice or more than twice a day significantly reducing D of teeth, D and DF of roots, MT, and the risk of root caries. Electric toothbrushing once or more than once a month significantly reduced D of crowns, roots, and teeth, DMFT, and the risk of caries in crowns, roots, and teeth while increasing the likelihood of DF of crowns and roots >0 and DFT >0. Dental cleaning with toothpicks substantially reduced MT and increased the risk of crown caries. Cleaning with toothpicks 1/month to 1/day significantly increased the risk of tooth caries, while cleaning with toothpicks ≥ 2/day significantly reduced DMFT. Dental diagnosis and treatment significantly increased F of crowns, MT, and DMFT while reducing the likelihood of F of teeth>0.

## Discussion

Oral diseases are gradual and cumulative, becoming more complex over time [1]. Older individuals commonly suffer from various oral diseases and face numerous barriers to accessing dental care services [11]. Compared to younger individuals, middle-aged and older adults are at a greater risk for active caries and periodontal diseases [11]. Oral diseases and disorders can considerably affect general health, well-being, and quality of life [1]. Improving oral health can boost the confidence of older patients, enable positive social activities, and restore their abilities to work [1].

Caries can be categorized into three types based on their location [12]: coronal, root, and a combination of both. They are caused by the shift from a dynamic balance to a metabolic imbalance in the ecosystem of acidogenic and aciduric bacteria that form the dental plaque biofilm [13]. Caries occurs as a result of the combination of biological and environmental processes on the tooth surface [14]. Ecological processes are influenced by behaviors, background, and social factors, which collectively affect the development of caries in individuals and populations through a series of steps [14]. However, the process of caries is very complex in real life [14], involving intricate interactions in the oral cavity, and individual and societal behaviors [14].

The results of this survey study highlight the differences in caries prevalence by gender, as shown in Tab 1. The main factors in caries formation are the presence of bacteria, the bacterial substrate (food/sugar), the oral environment of hosts, and the passage of time [15]. Risk factors for dental caries in women include different salivary composition and flow, hormonal fluctuations, dietary habits, genetic variations, and specific social roles in families[15]. Hormonal fluctuations in women tend to result in a less protective salivary composition and flow rate [15], leading to a greater susceptibility to caries.

Age, whether as a confounding factor or a direct determinant, is a critical variable in the diagnosis, etiology, and intervention research of caries and periodontal diseases [8]. As with many diseases, age significantly influences the prevalence of caries and periodontal diseases more than other known risk factors and can explain variations in occurrence [12]. The average gingival recession increases in older adults aged 65–74 and those over 75 [16], making them more susceptible to root caries. Biological changes due to aging make older individuals more susceptible to diseases and less adaptive to injuries [8]. With aging, the D and DF of roots in individuals with root caries and root fillings due to root caries increased significantly; however, single-factor epidemiological cross-sectional surveys showed no significant difference between the high and low age groups for the two indicators. The role of age in caries is attributed to cumulative exposure [12]. The "age-related susceptibility" hypothesis suggests that as age increases, dysregulation of the immune system or "immunosenescence" increases the risk of periodontal disease [17]. However, the connection between disease and age is complex [8]. For many diseases, including oral diseases, the variance in incidence explained by age appears to be greater than that explained by other known variables [8].

Due to the cumulative destruction of periodontal tissues, the surfaces susceptible to caries increase [8]. The relative importance of caries and periodontal disease as driving factors for tooth loss varies across different age groups [18]. Among children and adolescents, due to the very low prevalence of periodontitis, caries is the most substantial single disease, causing tooth loss [19]. In adults, caries and periodontitis are the primary reasons for tooth loss; however, the relative contribution of each disease varies significantly in different studies [19]. Variations in the distribution of caries and periodontal diseases have led to a significant increase in teeth retained by older adults, resulting in more tooth surfaces being exposed to root caries [8]. Exposed tooth roots and poor plaque control create a favorable environment for root surface caries [20]. Measures of caries exposure are related to periodontal status, and these associations may be population-specific [18]. DMFT tends to increase with the severity of periodontitis in the same participants [21]. Individuals with periodontal pockets are at significantly increased risk for root caries; those with dental calculus had significantly higher D of crowns, roots, teeth, and DF of roots. However, the prevalence rates of DFT, MT, and DMFT were reduced significantly, the risk of coronary and tooth caries substantially increased, and the risk of coronal DF was considerably reduced. There is a continued long-term trend of the main determinants of caries and periodontitis in older adults [8]. However, to date, the relationship between caries and periodontal disease remains controversial [12]. No significant association has been identified between periodontitis and the experience of root caries [20]. This discrepancy is possibly because *Streptococcus mutans* is a primary cariogenic bacterium, while periodontitis is associated with specific Gram-negative anaerobes like *Porphyromonas gingivalis *[12]. Reasonable plaque control can mitigate the risk factors of exposed tooth root surfaces [20].

Global studies have shown differences in oral health knowledge, beliefs, and practices between urban and rural populations [22]. Urban participants report more positive oral health beliefs and an in-depth understanding of oral disease prevention than rural participants [22]. Moreover, the percentage of toothpaste non-users is higher among rural participants [22]. This disparity may also be due to accessibility issues in rural areas[22]. Rural areas generally have fewer dentists per capita and higher poverty levels [23]. However, compared to rural individuals, urban residents consume sugary foods more frequently [22]. Over the past 30 years, the prevalence and incidence of caries have declined across all age groups in many regions. However, not all social groups have equally benefited from this decrease [19]. The overall dental filling status of urban residents is better than that of rural residents; however, the prevalence of root caries is higher among urban residents compared to those in rural regions. Living conditions significantly affect dental care behaviors among middle-aged and older individuals [11]. There are several explanations for these disparities [11]. In China, medical insurance coverage is higher in urban areas [11]. In rural areas, most middle-aged and older participants pay for dental healthcare themselves, indicating that low coverage of dental insurance directly impacts their dental care-seeking behavior [11]. Furthermore, the frequency of regular oral health check-ups and periodontal treatments is higher in urban areas [11]. Urban populations have a higher perceived need for dental care than rural populations [11]. Educational level plays a significant role in oral health. Higher education levels are associated with a higher F of teeth and a lower occurrence of root caries. For individuals with DF of roots, higher education correlates with DF of teeth; those with DFT show a significant increase in DFT. There is no significant association between educational level and D of teeth, DFT, MT, DMFT, and root caries rate for individuals with high school or higher education. There is a clear trend indicating that the preventive role of education against tooth loss increases over time, and the inequality in tooth loss prevalence due to educational level decreases gradually [24].

Caries are considered a diet-mediated disease because sugar plays a crucial role in their formation [25]. Individuals who consume snacks and candies at a frequency of once or more than once a week experience a significant increase in root caries if they already have root caries, and the prevalence of developing root caries also increases substantially. Those who regularly consumed sweet drinks were at a significantly higher risk of developing crown and root caries or exacerbating existing caries. Single-factor cross-sectional epidemiological analyses indicated a positive correlation between the frequency of consuming sugary drinks and the prevalence of crown and root caries. Logistic regression models have also shown that respondents were more likely to develop dental caries if they ingested candy or soft drinks more than once a day or occasionally within a week [22]. Changes in the oral environment associated with lifestyle and ecological alterations in the oral microbiota may lead to changes in biofilms’ internal structure and function, disrupting the balance and resulting in microbial imbalance [13]. Unfavorable oral conditions, elevated levels of pathogenic bacteria, and the fermentation rate of carbohydrates may alter the balance of demineralization and remineralization cycles, favoring the development of caries [26]. Disruption of the typical balance and periodic demineralization and remineralization processes within biofilms can initiate and progress caries [27]. Smoking is recognized as a risk factor for dental caries formation [28]. It is associated with an increased risk of dental caries[29], accelerates aging, and weakens the immune system [30]. Smoking increases the susceptibility of older individuals to infections and exacerbates existing systemic diseases [30]. Prolonged tobacco exposure can cause significant changes in the microbiota, leading to dysbiosis of the oral flora [31] and predisposing individuals to dental caries. Cigarette components promote the growth of cariogenic microorganisms [31]. Nicotine enhances the activity of *Streptococcus pyogenes*, *Lactobacillus*, *Streptococcus gordonii, Actinobacillus,* and *Candida albicans *[31]. The symbiotic bacterium *Streptococcus haematobium* shows lower competitiveness in the presence of nicotine [31]. *Streptococcus pyogenes* isolated from smokers are more susceptible to high nicotine concentrations compared to non-smokers [32]. Smoking affects saliva by reducing its buffering capacity, and altering its chemical and bacterial composition, thereby promoting a caries-prone environment [31]. Secretory immunoglobulin A (sIgA) is the predominant immunoglobulin in saliva and is the primary specific defense mechanism in the oral cavity [33]. sIgA, along with various antimicrobials (such as lysozyme, lactoferrin, salivary peroxidase, and visfatin), limits microbial adhesion to epithelial and tooth surfaces, thereby helping to prevent oral diseases [34–36]. Smokers have a higher prevalence of dental caries and lower sIgA concentrations than non-smokers [33]. Reduced sIgA levels correlate with an increased prevalence of dental caries [33]. Smoking protects against the risk of crown, root, and tooth caries. However, there is limited evidence regarding the relationship between smoking and caries, and a review suggested that the link between smoking and increased caries risk is weak [29]. However, healthcare providers should strive to motivate older individuals to quit smoking [30].

This study could not identify a correlation between systemic diseases and caries. Multivariate logistic regression analysis has shown that adults with diabetes who had caries were more likely to develop additional caries compared to those without diabetes [37]. However, significant differences have not been observed in the prevalence of caries based on diabetes status [38]. Further in-depth research is needed to understand the causal relationship between diabetes and caries.

Caries and periodontal diseases are the adults’ most common oral health issues [12]. Toothbrushing is widely recognized as a simple, inexpensive, and effective method to reduce the occurrence of these conditions. Increased frequency of manual and electric toothbrushing is associated with significant reductions in the D of crowns, roots, teeth, and the risk of root caries. Moreover, as the frequency of electric toothbrushing increases, the risk of crown and tooth caries decreases. Tooth loss is a critical indicator of oral and overall health [24]. The frequency of manual toothbrushing and the use of toothpicks for cleaning teeth were negatively correlated with MT. Higher frequencies of teeth cleaning with manual or electric toothbrushes and toothpicks significantly reduce DMFT. The frequency of toothbrushing is significantly associated with the number of caries in permanent teeth, suggesting that caries rates are more influenced by oral health behaviors [23]. However, it is essential to consider the frequency and quality of toothbrushing when examining the relationship between caries and oral hygiene [39]. Using toothpicks to clean teeth at a frequency of once per month to once per day increases the caries risk. Given the limited research on the correlation between toothpick use and caries, a specific causal relationship cannot be determined. Economic status is a significant factor that limits access to dental diagnosis and treatment [11]. This study found that diagnosis and treatment behaviors considerably increased the occurrence of F of crowns, MT, and DMFT while reducing the prevalence of F of teeth. Developing a healthy lifestyle, practicing appropriate self-care, and regularly using oral health services when available are recommended [40].

The 8020 program promotes the retention of at least 20 natural teeth by age 80 [41], aiming to improve overall health and quality of life. People who achieve this goal tend to be healthier, suffer from fewer diseases, and lead more comfortable lives than those who do not [41]. This initiative has become a national mandate based on the belief that having more natural teeth can significantly enhance one's quality of life, particularly in old age [41]. However, collecting data from 80-year-olds with 20 or more natural teeth and studying their health remains challenging for researchers seeking to improve the quality of life in older individuals [41]. This study has some limitations. Due to the complexity of the survey and the numerous influencing factors that needed to be analyzed, the factors included were generally broad. Details such as the severity of diabetes, average toothbrushing time per session, and specific reasons for dental visits were not included, making it difficult to draw precise conclusions. Moreover, the differences in sample sizes between some variable groups (such as the use of electric toothbrushes, dental floss, and water flossers) may lead to statistical results that do not fully reflect objective realities.

## Conclusions

In Guangdong Province, the prevalence of coronary and root caries among older individuals aged 65–74 years remains severe. Dental health service workers should guide older individuals to limit their intake of sweets and sugary drinks, teach them proper dental cleaning techniques, and encourage a consistent toothbrushing routine (at least twice a day). It is essential to treat periodontal diseases promptly and regularly monitor the caries status of the older individuals in their care. Medical insurance institutions should increase economic assistance to diagnose and treat caries, particularly for older adults in rural areas.

## Data Availability

The datasets generated and/or analysed during the current study are not publicly available due to the information that could compromise participants’ privacy but are available from the corresponding author on reasonable request.
